# Relationship between *Trypanosoma brucei rhodesiense* genetic diversity and clinical spectrum among sleeping sickness patients in Uganda

**DOI:** 10.1186/s13104-017-2860-x

**Published:** 2017-10-27

**Authors:** Charles D. Kato, Claire M. Mugasa, Ann Nanteza, Enock Matovu, Vincent P. Alibu

**Affiliations:** 1 School of Bio-security, Biotechnical & Laboratory Sciences, College of Veterinary Medicine, Animal Resources & Bio-security, Makerere University, P.O Box 7062, Kampala, Uganda; 2 College of Natural Sciences, Makerere University, P.O Box 7062, Kampala, Uganda

**Keywords:** Human African trypanosomiasis, Sleeping sickness, Clinical diversity, Genetic diversity, Multi-locus genotypes, Microsatellite markers

## Abstract

**Objective:**

Human African trypanosomiasis (HAT) due to *Trypanosoma brucei rhodesiense* in East and southern Africa is reported to be clinically diverse. We tested the hypothesis that this clinical diversity is associated with a variation in trypanosome genotypes.

**Results:**

Trypanosome DNA isolated from HAT patients was genotyped using 7 microsatellite markers directly from blood spotted FTA cards following a whole genome amplification. All markers were polymorphic and identified 17 multi-locus genotypes with 56% of the isolates having replicate genotypes. We did not observe any significant clustering between isolates and bootstrap values across major tree nodes were insignificant. When genotypes were compared among patients with varying clinical presentation or outcome, replicate genotypes were observed at both extremes showing no significant association between genetic diversity and clinical outcome. Our study shows that *T. b. rhodesiense* isolates are homogeneous within a focus and that observed clinical diversity may not be associated with parasite genetic diversity. Other factors like host genetics and environmental factors might be involved in determining clinical diversity. Our study may be important in designing appropriate control measures that target the parasite.

**Electronic supplementary material:**

The online version of this article (10.1186/s13104-017-2860-x) contains supplementary material, which is available to authorized users.

## Introduction

Human African trypanosomiasis (HAT) or sleeping sickness is caused by extra-cellular protozoan parasites *Trypanosoma brucei rhodesiense* (East and southern Africa) and *Trypanosoma brucei gambiense* (West and central Africa). Previously, HAT due to *T. b. rhodesiense* has been classified as an acute disease with rapid progression [1, 2]. It is now clear that *T. b. rhodesiense* disease is clinically diverse, both within and across foci [3–8]. This observed clinical diversity has been partly attributed to a variation in trypanosome strains [9]. A high degree of genetic diversity has been demonstrated among *T. brucei* stocks using highly polymorphic microsatellite markers [5, 10, 11]. Laboratory studies have shown that the parasite might be a strong driver for HAT associated pathology. When differences in pathology were compared in a mouse model using two distinct *T. b. brucei* strains (TREU-927 and STIB-247), mice infected with strain TREU-927 suffered a more severe clinical disease [12].

In a study comparing HAT clinical diversity among patients in Uganda and Malawi, patients in Uganda suffered a more severe disease [6]. When parasite genotypes involved were analyzed, two sequence variants of the serum resistance antigen (SRA) gene were observed. However, these results were not conclusive since the SRA gene is not polymorphic to infer genetic diversity. When polymorphic microsatellite markers were used to compare disease in a restricted geographical area in Uganda (Tororo and Soroti), two distinct genotype clusters with varying disease response were observed [5]. However, all these studies compared parasite genotypes between different HAT foci. With the observed clinical diversity within a localized HAT foci [3, 7] it is likely that the parasite might have a role to play.

We hypothesized that parasite genotypes within a single HAT focus are diverse. We genotyped trypanosomes directly from blood spotted FTA cards using 7 microsatellite markers following whole genome amplification. We further associated parasite genotypes to observed clinical diversity.

## Main text

### Materials and methods

#### Study design

Patients were recruited passively at Lwala hospital in northern Uganda from the year 2012–2014. Routine diagnosis of suspected HAT patients, was done by microscopic examination of wet and thick blood films [13]. For inclusion in the study, patients needed to have a positive blood smear or those with a negative blood smear but with signs indicative of HAT with trypanosomes demonstrated in cerebrospinal fluid (CSF) or an elevation in CSF white blood cell count [14]. Exclusion criteria were: terminally ill patients, children below 6 years and patients whose disease stage could not be ascertained. Physical examination was done by a medical officer and on each patient clinical data form demographic characteristics, signs and symptoms of HAT were recorded.

#### FTA card preparations and whole genome amplification

From each patient, approximately 200 µl of blood was spotted on the FTA card (Whatman). Whole genome amplification (WGA) was performed using the Ready-To-Go Genomiphi V3 DNA amplification kit (GE Healthcare, Sweden) as described previously [15, 16]. Briefly, 20 µl of cell lysis solution (400 mM KOH, 10 mM EDTA, 100 mM DTT) were added to 2-mm FTA discs. For WGA, 20 µl of the denatured cell lysate DNA was added to the Genomiphi V3 cake and samples incubated at 30 °C for 2 h followed by heating at 65 °C for 10 min with subsequent cooling at 4 °C. Three independent WGA reactions from the same sample were pooled and stored at − 20 °C until further use.

#### Polymerase chain reaction based genotyping and multi-locus genotype determination

We used 7 previously described microsatellite loci, Ch1/18, Ch2/5, Ch2/PLC, Ch4/M12C12, Ch3/5L5, Ch5/JS2 [5, 10, 17, 18] and M6C8 [19, 20] as shown in Additional file 1. All PCRs were performed in a final volume of 20 µl, containing: PCR buffer (50 mM tris-HCl (pH 9.0), 50 mM NaCl, 0.1 mg/ml BSA and 5 mM MgCl_2_), 200 µM of each dNTPs, 10 ng gDNA, 1 µm of forward and reverse primer and 1 unit of EconoTaq DNA polymerase (Lucigen, USA). PCR amplification conditions were, an initial denaturation at 95 °C for 3 min, followed by 45 cycles of 30 s at 95 °C, 30 s at 60–55 °C and a final elongation step for 20 min at 72 °C.

Allele size determination was done using a capillary based sequencer, the 3500xL Genetic Analyzer (Applied Biosystems) using GeneMapper Software v5.0 (Life Technologies). Multi-locus genotypes (MLGs) were defined by the specific combination of alleles across all loci (see Additional file 2).

#### Genetic and statistical analyses

We analyzed genetic data on allele frequencies, heterozygosity, allelic richness, and inbreeding coefficient (F_IS_) using GenAIEx v6.5 [21]. A dendrogram based on MLGs was constructed using the neighbor-joining method in MEGA v6 [22]. Data on clinical diversity was analyzed using IBM SPSS version 22. Numerical variables were summarized using medians. Univariate analysis to compare clinical diversity between those with the condition and those without was done using cross-tabulation with a Chi square or Fisher’s exact test.

### Results

#### Patient’s baseline characteristics and clinical diversity

A total of 25 HAT cases were recruited passively at Lwala hospital in Northern Uganda. A significant (P < 0.001) number of patients were diagnosed as late stage (18, 75%) compared to early stage patients (6, 25%). The male: female ratio for the patients was 2, with a median age of 23.8 years (Table 1). The observed clinical spectrum is presented in Table 1.

**Table 1:** Patient’s baseline characteristics

Characteristic	Early stage	Late stage	P value
Disease stage	6	18 (75%)	< 0.001*
Sex (male/female)	5/1	11/7	0.319
Age (median)	17.8	24.5	0.444
Trypanosomes in CSF	0	16 (66.7%)	< 0.00*
Parasitemia/ml	19.2 × 10^4^	36.1 × 10^4^	0.399
CSF parasitosis/ml	0	8.0 × 10^4^	< 0.01*
Disease duration (median)	0.5	1.3	0.173
Clinical presentation			
Fever	6	17 (100%)	1.0
Headache	6	16 (94.1%)	0.417
Hepatomegaly	0	2 (8.7%)	0.538
Lymphadenopathy	2	6 (26.1%)	0.666
Splenomegaly	0	6 (26.1%)	0.123
Edema	0	4 (17.4%)	0.232
Somnolence	1	8 (34.8%)	0.208
Gait abnormalities	0	6 (26.1%)	0.123
Tremors	0	2 (8.7%)	0.538
Urinary incontinence	0	2 (8.7%)	0.538
Cranioneuropathy	0	2 (9.1%)	0.519
Reactive encephalopathy	0	1 (4.3%)	0.739
Glasgow coma score			
Mild (13–150)	6	12 (54.5%)	0.217
Moderate (9–12)	0	3	
Severe (≤ 8)	0	1	

* Significantly higher in late stage patients

#### Microsatellite marker validation

We genotyped 25 trypanosome positive samples using 7 microsatellite markers. Marker Ch5/JS2 was monomorphic across all samples and was removed from further analysis. All markers except M6C8 exhibited an excess of heterozygosity (Ho > He, F_IS_ < 0) with marker Ch4/M12C12 tending to heterozygosity fixation (F_IS_ = − 1.00, Additional file 3). When allele frequencies were analyzed, marker Ch1/18 was the most polymorphic with 6 alleles in total, followed by Ch2/5 (4 alleles), Ch3/5L5/2 (3 alleles) the remaining markers had only 2 alleles each (Additional file 4).

#### Genetic diversity

**Fig. 1:**
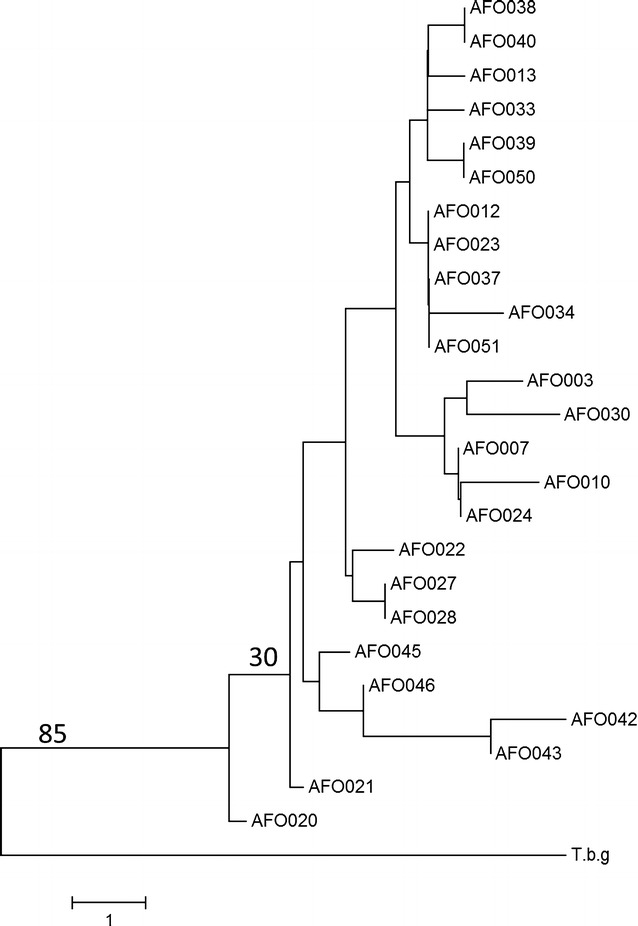
Neighbor-joining dendrogram showing genetic similarity among *T. b. rhodesiense* isolates. *T.b.g* is a *T. b. gambiense* isolate included for comparison

Among the 25 genotyped samples, a total of 17 multi-locus genotypes (MLGs) were identified. Among the 17 MLGs, 44% (11/25) of the samples were associated with unique genotypes. Replicated genotypes were found in 56% (14/25) of the genotyped samples. MLG 4 occurred 4 times (16%) while all the other repeated MLGs were encountered only twice (8%). To demonstrate the relationship between *T. b. rhodesiense* isolates, we constructed a neighbor-joining tree using pairwise distance between the MLGs (Fig. 1). We did not observe any significant clustering across isolates and bootstrap values across major nodes were insignificant.

#### Correlation between clinical and genetic diversity

We carried out an evaluation to associate the scored MLGs with observed clinical diversity. However, due to the high heterogeneity of MLGs and the low power of the study, meaningful statistics could not be done. We compared the distribution of MLGs among patients with varying clinical presentation or outcome (Table 2). We did not observe any significant association between MLGs and clinical outcome. When genotypes were compared among patients with varying degree of neurological impairment as a measure of disease severity, MLGs 4, 13 and 14 were common in all extremes. Furthermore, when we compared clinical presentation between those with and without the clinical manifestation, repeated genotypes were observed at both extremes.

**Table 2:** Relationship between clinical outcome and multi-locus genotypes

Characteristic	Frequency	Associated MLGs
Severity of neurological response		
Mild (13–15)	19 (82.6%)	1,2,3,*4*,5,6,7,8,9,10,12,*13*,*14*,15,16,17
Moderate (9–12)	3	11,*13*,*14*
Severe (≤ 8)	1	*4*
Disease duration (months)		
< 1	14 (63.6%)	*4*,5,9,12,13,14,16,17
1–3	7	1,2,3,6,8,10,11
> 3	1	*4*
Clinical presentation		
Fever		
Yes	24 (100%)	1,2,3,4,5,6,7,8,9,10,11,12,13,14,16,17
No	0	
Headache		
Yes	22 (91.7%)	1,2,3,4,5,7,8,*9*,10,11,12,13,14,15
No	2	*9*,17
Hepatomegaly		
Yes	2 (8.3%)	*4*,5
No	22	1,2,3,*4*,6,7,8,9,10,11,12,13,14,15,16,17
Lymphadenopathy		
Yes	8 (33.3%)	2,4,9,10,*13*,*14*,*15*
No	16	1,3,4,5,6,7,8,9,11,12,*13*,*14*,*15*,16,17
Splenomegaly		
Yes	6 (25%)	*4*,5,*9*,*13*,*14*,16
No	18	1,2,3,*4*,6,7,8,*9*,10,11,12,*13*,*14*,15,17
Edema		
Yes	4 (16.7%)	*4*,*13*,16
No	20	1,2,3,*4*,5,5,6,7,8,9,10,11,12,*13*,14,15,17
Somnolence		
Yes	9 (37.5%)	1,*2*,3,5,6,8,9,10,12
No	15	*2*,4,7,11,13,14,15,16,17
Gait abnormalities		
Yes	6 (25%)	1,*4*,*13*,*9*,11
No	18	2,3,*4*,5,6,7,8,*9*,10,12,*13*,14,15,16,17
Tremors		
Yes	2 (8.3%)	11,*15*
No	22	1,2,3,4,5,6,7,8,9,10,12,13,14,*15*,16,17
Urinary incontinence		
Yes	2 (8.3%)	*4*,11
No	22	1,2,3,*4*,5,6,7,8,9,10, 12,13,14,15,16,17
Cranioneuropathy		
Yes	3 (15%)	14,*15*
No	20	1,2,3,4,5,6,7,8,9,10,11,12,13,14,*15*,16

Multi-locus genotypes indicated in italics were present in those with and without the clinical sign or across groups

### Discussion

Sleeping sickness due to *T. b. rhodesiense* is described as clinically diverse, with varying disease severity and duration of illness across foci [4–8]. Similarly, a wide disease spectrum within the same HAT foci has been reported [3, 7] albeit, with reduced frequency. We tested the hypothesis that this variation in clinical diversity is associated with diversity in parasite genotypes.

In this study, we identified 17 multi-locus genotypes and among these, replicate genotypes were found in 56% of the isolates. With more than half of the samples constituted by repeated genotypes and the low bootstrap support for the phylogenetic tree, *T. b. rhodesiense* isolates in this focus appear homogenous with limited genetic diversity. Our findings are in agreement with a previous study in Soroti were *T. b. rhodesiense* isolates were homogeneous with replicate genotypes constituting 59% of the population [10]. Indeed, when isolates from 2 related HAT foci in Uganda (Tororo and Soroti) were compared, the 2 populations could not be resolved with confidence due to the similar genotypes involved. This limited genetic diversity is consistent with previous studies describing *T. b. rhodesiense* stocks in Uganda as strictly clonal [10, 23].

In previous studies, a link between clinical diversity and parasite genetic diversity was proposed [9]. We investigated this possibility using our data set. However, we did not observe any significant associations between genotypes and clinical outcome. In almost all cases replicate genotypes were found at all extremes of clinical outcome. Our results are in agreement with a previous *T. b. gambiense* study in Cote d’Ivoire that did not find any correlation between clinical diversity and parasite genotype [24]. Similar to our study, the genotypes isolated from the latter study were homogeneous with limited genetic variation. Furthermore, when two zymodemes with varying clinical phenotypes (Zambezi and Busoga strains) were compared, the 2 strains were extremely similar [25]. However, studies comparing *T. b. rhodesiense* isolates across different HAT foci associated clinical outcome to distinct parasite genotypes [5, 6, 8]. With the observed, genotypic homogeneity within a single HAT focus in this study, our results are expected. Due to the high polymorphic nature of microsatellite markers, a high number of MLGs appear even with minimal genetic differentiation. Indeed, with the high mutation rates within microsatellite loci [26, 27] related genes might be seen as slightly different [28] without necessarily changing virulence. Thus, although these markers have been shown to be perfect markers for inferring population structure, they pose a challenge in associating parasite diversity to clinical outcome.

### Conclusion

Our study shows that *T. b. rhodesiense* isolates are homogeneous within a focus and that observed clinical diversity may not be related to parasite genetic diversity. It is likely that other factors like host genetic polymorphism and unknown environmental factors might be involved in driving clinical diversity. Due to the limited number of patients in this preliminary study, research studies recruiting large patient groups from different geographical locations are recommended for more conclusive results.

### Limitations of the study

As a result of the low incidence of disease, we were unable to achieve a sample size greater than 25 patients. Further, this small sample size allowed us to compare only 18 late stage patients and 6 early stage patients. Consequently, this limited sample could not allow more conclusive statistics to be done. Indeed, correlation studies between observed genotypes and clinical presentation would have benefited from a larger sample size so as to cater for both disease extremes. To try and make the study more conclusive, we used a total of 7 markers so as to pave way for further investigations.

## Additional files

**Additional file 1.** Microsatellite loci and primer sequences.

**Additional file 2.** Multi-locus genotypes (MLGs) and allele sizes for 7 microsatellite markers.

**Additional file 3.** Heterozygosity and polymorphism within the population.

**Additional file 4.** Allele frequencies across the different microsatellite loci.

## Abbreviations

CSF: cerebrospinal fluid
GCS: glasgow coma scale
HAT: human African trypanosomiasis
MLGs: multi-locus genotypes
PCR: polymerase chain reaction
SRA: serum resistance antigen
WBC: white blood cell
WGA: whole genome amplification
