# Supplementary material for: Relationship between Trypanosoma brucei rhodesiense genetic diversity and clinical spectrum among sleeping sickness patients in Uganda
Source: BMC Res Notes. 2017 Oct 27;10:518. doi: 10.1186/s13104-017-2860-x (PMC5658916; doi:10.1186/s13104-017-2860-x)
Supplement: Supplementary file 2 — Additional file 2. Multi-locus genotypes (MLGs) and allele sizes for 7 microsatellite markers. [file 13104_2017_2860_MOESM2_ESM.docx]

**Additional file 2:** Multi-locus genotypes (MLGs) and allele sizes for 7 microsatellite markers

| Sample | Sex | Ch1/18 | Ch2/5 | Ch3/5L5/2 | Ch4/M12C12 | Ch5/JS2 | Ch2/PLC | M6C8 | MLG |
| --- | --- | --- | --- | --- | --- | --- | --- | --- | --- |
| AFO003 | M | 180/219 | 104/142 | 171/173 | 123/128 | 100/100 | 157/181 | 094/094 | 1 |
| AFO007 | M | 180/219 | 142/142 | 171/173 | 123/128 | 100/100 | 157/181 | 094/094 | 2 |
| AFO010 | F | 180/219 | 142/142 | 171/173 | 123/128 | 100/100 | 181/181 | 094/094 | 3 |
| AFO012 | M | 180/219 | 142/142 | 173/173 | 123/128 | 100/100 | 157/181 | 094/094 | 4 |
| AFO013 | M | 180/343 | 142/142 | 173/173 | 123/128 | 100/100 | 157/181 | 094/094 | 5 |
| AFO020 | M | 180/219 | 108/142 | 171/173 | 123/128 | 100/100 | 157/181 | 075/094 | 6 |
| AFO021 | M | 180/219 | 142/142 | 171/173 | 123/128 | 100/100 | 157/181 | 075/094 | 7 |
| AFO022 | M | 180/360 | 142/142 | 173/173 | 123/128 | 100/100 | 157/181 | 075/094 | 8 |
| AFO023 | M | 180/219 | 142/142 | 173/173 | 123/128 | 100/100 | 157/181 | 094/094 | 4 |
| AFO024 | M | 180/219 | 142/142 | 171/173 | 123/128 | 100/100 | 157/181 | 094/094 | 2 |
| AFO027 | M | 180/298 | 142/142 | 173/173 | 123/128 | 100/100 | 157/181 | 075/094 | 9 |
| AFO028 | F | 180/298 | 142/142 | 173/173 | 123/128 | 100/100 | 157/181 | 075/094 | 9 |
| AFO030 | M | 180/180 | 120/142 | 171/173 | 123/128 | 100/100 | 157/181 | 094/094 | 10 |
| AFO033 | F | 180/180 | 142/142 | 173/173 | 123/128 | 100/100 | 157/181 | 094/094 | 11 |
| AFO034 | F | 180/219 | 142/142 | 173/173 | 123/128 | 100/100 | 157/157 | 094/094 | 12 |
| AFO037 | F | 180/219 | 142/142 | 173/173 | 123/128 | 100/100 | 157/181 | 094/094 | 4 |
| AFO038 | F | 180/356 | 142/142 | 173/173 | 123/128 | 100/100 | 157/181 | 094/094 | 13 |
| AFO039 | M | 180/298 | 142/142 | 173/173 | 123/128 | 100/100 | 157/181 | 094/094 | 14 |
| AFO040 | F | 180/356 | 142/142 | 173/173 | 123/128 | 100/100 | 157/181 | 094/094 | 13 |
| AFO042 | M | 180/219 | 142/142 | 173/173 | 123/128 | 100/100 | 157/157 | 075/075 | 15 |
| AFO043 | M | 180/219 | 142/142 | 173/173 | 123/128 | 100/100 | 157/181 | 075/075 | 15 |
| AFO045 | F | 180/219 | 142/142 | 173/183 | 123/128 | 100/100 | 157/181 | 075/094 | 16 |
| AFO046 | F | 180/219 | 142/142 | 173/173 | 123/128 | 100/100 | 157/181 | 075/094 | 17 |
| AFO050 | M | 180/298 | 142/142 | 173/173 | 123/128 | 100/100 | 157/181 | 094/094 | 14 |
| AFO051 | M | 180/219 | 142/142 | 173/173 | 123/128 | 100/100 | 157/181 | 094/094 | 4 |
|  |  |  |  |  |  |  |  |  |  |

Results are given as AA/BB, where AA is the size (base pairs) of the small allele and BB is size of the larger allele.
