# Supplementary material for: Relationship between Trypanosoma brucei rhodesiense genetic diversity and clinical spectrum among sleeping sickness patients in Uganda
Source: BMC Res Notes. 2017 Oct 27;10:518. doi: 10.1186/s13104-017-2860-x (PMC5658916; doi:10.1186/s13104-017-2860-x)
Supplement: Supplementary file 3 — Additional file 3. Heterozygosity and polymorphism within the population. [file 13104_2017_2860_MOESM3_ESM.docx]

**Additional file 3**: Heterozygosity and polymorphism within the population

| Loci | Na | Ne | Ho | He | F_IS_ |
| --- | --- | --- | --- | --- | --- |
| Ch1/18 | 6 | 2.56 | 0.92 | 0.61 | -0.51 |
| Ch2/5 | 4 | 1.13 | 0.12 | 0.11 | -0.04 |
| Ch3/5L5/2 | 3 | 1.38 | 0.32 | 0.27 | -0.17 |
| Ch4/M12C12 | 2 | 2.00 | 1.00 | 0.50 | -1.00 |
| Ch2/PLC | 2 | 2.00 | 0.88 | 0.50 | -0.76 |
| M6C8 | 2 | 1.52 | 0.28 | 0.34 | 0.18 |
|  |  |  |  |  |  |

Na: number of alleles, Ne: number of effective alleles, Ho: observed heterozygosity, He: expected heterozygosity, F_IS_: inbreeding coefficient
