# Supplementary figures and images for: Relationship between Trypanosoma brucei rhodesiense genetic diversity and clinical spectrum among sleeping sickness patients in Uganda
Source: BMC Res Notes. 2017 Oct 27;10:518. doi: 10.1186/s13104-017-2860-x (PMC5658916; doi:10.1186/s13104-017-2860-x)

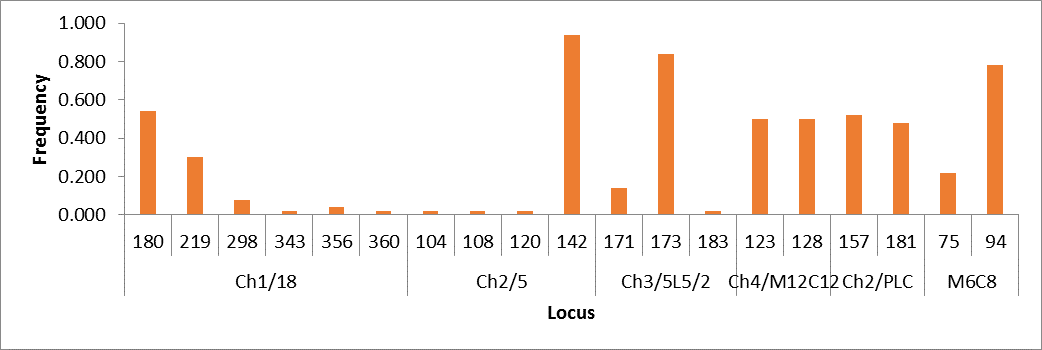

Supplement: Supplementary file 4 — Additional file 4. Allele frequencies across the different microsatellite loci. [file 13104_2017_2860_MOESM4_ESM.png]
